# Utility of* Borrelia burgdorferi* sensu stricto C6 Peptide for Serologic Confirmation of Erythema-Free Ixodid Tick-Borne Borrelioses in Russia

**DOI:** 10.1155/2018/5291926

**Published:** 2018-06-28

**Authors:** Vera Pomelova, Eduard Korenberg, Tatiana Kuznetsova, Nikolay Osin

**Affiliations:** ^1^Laboratory of Molecular Diagnostics, Department of Biological Microassay, State Research Institute of Biological Engineering, 75/1 Volokolamskoye Highway, Moscow 125424, Russia; ^2^Department of Infections with Natural Focality, N.F. Gamaleya Research Institute of Epidemiology and Microbiology, 18 Gamaleya Street, Moscow 123098, Russia; ^3^Laboratory of Clinical Immunology, Perm Krai Clinical Infectious Diseases Hospital, 96 Pushkin Street, Perm 614000, Russia; ^4^Immunoscreen Closed Joint Stock Company, 75/1 Volokolamskoye Highway, Moscow 125424, Russia

## Abstract

We evaluated the utility of* Borrelia burgdorferi *sensu stricto* (Bb)* peptide C6 for serologic confirmation of Ixodid Tick-Borne Borrelioses (ITBB) in Russia. Serum samples (N = 1089) were from erythema migrans (EM) (N = 327) and the EM-free (EMF) patients (N = 115); in some patients, the disease was accompanied by human granulocytic anaplasmosis or tick-borne encephalitis. The sera were investigated by multiplex phosphorescence analysis (PHOSPHAN) for IgM to* Bb* C6, recombinant OspC and VlsE proteins, and IgG to C6 from* Bb*,* B. garinii* (*Bg*), and* B. afzelii* (*Ba*). Detection of* Bb* C6 IgM/IgG provided effective serologic confirmation of ITBB in both EM and EMF patients early after disease onset. In the EM-free patients, however, this test needed to be supplemented with detection of VlsE IgM in convalescent-phase sera due to delay in development of the antibody responses for C6 IgG. In general, positive PHOSPHAN reactions were observed in 81.9% and 86.7% of the EM and EMF patients, respectively, as well as in 59 of 65 (90.8%) patients, whose blood contained* B. burgdorferi* sensu lato DNA. Additional detection of IgG to* Bg* C6 or* Ba* C6 had no significant contribution to serologic diagnosis of ITBB in both patient groups.

## 1. Introduction

Ixodid Tick-Borne Borrelioses (ITBB) are etiologically independent infections of the Lyme Borreliosis group in Russia [1]. They are caused by spirochetes of the* Borrelia burgdorferi *sensu lato group transmitted by ixodid ticks. Russia is the largest global areal of* B. burgdorferi* sensu lato [2, 3], with ITBB cases recorded in 74 out of 85 administrative regions. The number of confirmed ITBB cases in 2016 reached approximately 6100, which was equivalent to 4.18 cases per 100,000 population [4].

Clinical manifestations of Lyme Borreliosis in the United States are differed significantly from those in Eurasia [3, 5–7]. This is largely due to genetic heterogeneity of* Borrelia* that cause the disease [8].* B. burgdorferi* sensu stricto (hereinafter referred to as* B. burgdorferi* or* Bb*) is the major causative agent of Lyme disease in North America, with only a few cases recently linked to* Borrelia mayonii* [9]. In Europe, at least 5 species of* Borrelia* (*B. afzelii, B. garinii, B. burgdorferi*,* B. spielmani,* and* B. bavariensis*) can cause the disease. Other species (*B. bissetii*,* B. lucitaniae,* and* B. valaisiana*) are sometimes identified in patients but not considered important pathogens [3]. On the territory of Russia, the circulation and distribution of* B. garinii, B. afzelii, B. lucitaniae, B. valaisiana, B. burgdorferi*, and other species of* Borrelia *have been established [10]. Data were obtained that characterized the genetic polymorphism of* B. garinii *and* B. afzelii* [11, 12], the most widespread agents in Eurasia, which are etiologic agents of almost all disease cases in both Europe and Russia [3, 7]. The probability of human infections with several species of* Borrelia* (borrelial-borrelial infection) is rather high. The agents of other tick-borne diseases (e.g., tick-borne encephalitis virus or the human granulocytic anaplasmosis agent) can be transmitted after a tick bite, causing a variety of mixed infections in humans (for review [7]).

In Russia, the ITBB disease is registered in medical documentation as “ITBB with erythema migrans” (EM) and “ITBB without EM or EM-free ITBB” (EMF) [5]. Typical EM is the only pathognomonic sign of early ITBB in Russia. In EM patients, the ITBB disease affects the skin, connective tissue, and musculoskeletal, nervous, and cardiovascular systems [3, 5, 6, 13]. In the United States and Europe, EM occurs in 70-90% of patients while the extracutaneous manifestations are much less frequent [13]. In Russia, the EM-free forms of ITBB can be diagnosed in 20-45% of patients in the acute period of the disease [5, 14]. The disease in EMF patients manifests somewhat later (after tick bite), than in EM patients. The disease begins acutely with increasing temperature and development of a general infection syndrome, which is often followed by development of organ pathology affecting one or another system [5]. Common clinical manifestations of the erythema-free form of ITBB are neurologic (22%), arthromyalgic (15%), flu-like (12%), cardiovascular (8%), hepatic (4%), and regional lymphadenitis (1%); a mixed variant, characterized by a combination of several signs and symptoms, occurs most frequently (38%) [14]. Pronounced clinical polymorphism in the acute period of ITBB provides evidence for dissemination of the pathogen and generalization of infection.

Clinical diagnosis of ITBB in EMF patients is based on the history of a tick bite, presence of mentioned above clinical manifestations consistent with borreliosis [5, 14], and laboratory evidence of* B. burgdorferi* sensu lato infection. Confirmation of active infection consisted of amplification of* B. burgdorferi* sensu lato DNA/RNA in blood by species-specific PCR and detection of anti-borreliae immunoglobulin (Ig) M and G in acute- and/or convalescent-phase serum samples [5].

However, as it was recently discovered, the febrile and systemic symptoms in tick-exposed patients may be also caused by* Borrelia miyamotoi *[15–18], a spirochete classified in the relapsing fever group; the* B. miyamotoi* disease (BMD) may present with clinical manifestations mimicking those in EMF patients. Importantly, the two-tiered diagnostic protocols recommended for serologic confirmation of* B. burgdorferi* infection [19] were reactive for IgM (but not IgG) antibodies to* B. burgdorferi* in convalescent sera from PCR confirmed BMD cases [18]. Moreover, the FDA-approved C6 peptide ELISA, recently proposed as a potential alternative to conventional two-tier testing [20, 21], also was regularly positive on convalescent-phase serum samples from Northeastern American patients with* B. miyamotoi* infection [22]. According to Telford et al. [18], an IgM reactive sample by Lyme ELISA, but not confirmed by immunoblot, may represent a response to* B. miyamotoi* instead of* B. burgdorferi*. Importantly, the convalescent sera from BMD patients rarely demonstrate IgG reactivity to* B. burgdorferi* antigens [18].

With this in mind, we present here results of using a multiplex method of phosphorescence analysis (PHOSPHAN) (Immunoscreen, Russia) for the detection of* Bb* IgM and IgG to C6 peptide and of IgMs to OspC or VlsE proteins of* B. burgdorferi* sensu lato. A high efficiency of this method for serologic confirmation of ITBB in EM patients was demonstrated by us earlier [23]. The objective of this research was to compare the utility of PHOSPHAN method for the serologic diagnosis of ITBB in EM patients and the EM-free patients, in which the disease with ITBB occurred as monoinfection or was accompanied by coinfections with human granulocytic anaplasmosis (HGA) or tick-borne encephalitis (TBE). To our knowledge, this is the first work that considers the evolution of IgM and IgG antibody responses to the C6 peptide and OspC and VlsE proteins of* B. burgdorferi* sensu lato in erythema-free ITBB patients as well as in EM and EMF patients coinfected with the agents of HGA or TBE.

## 2. Materials and Methods

### 2.1. Ethics Statement

The research was performed under the State Research Institute of Biological Engineering, approved protocols numbers 2010/3135/11 and 2017/07/16. Case histories of the patients were deidentified prior to investigation, so the patient informed consent was not required.

### 2.2. Study Design

Serum samples (N = 1089) were collected from 442 patients with EM and the EM-free (EMF) extracutaneous forms of ITBB during the period of May to September of 2010-2011 in a highly endemic region (Perm Krai) located in the cis-Ural part of Russia. The patients were divided into 6 groups according to physician diagnosed clinical form of ITBB (EM or EMF) and the presence of coinfection with HGA or TBE agents. Serum samples from each group were tested separately for* Bb* C6 IgM and IgG; for OspC IgM and VlsE IgM; for* Bg* C6 IgG and* Ba* C6 IgG using the PHOSPHAN method. IgG antibody responses to recombinant proteins were not included in the analysis since they had no significant contribution to the total C6-IgG responses measured in both EM patients and EMF patients (as was shown in preliminary optimization experiments).

### 2.3. Case Definitions

The clinical diagnosis of ITBB in EM patients (N = 327) was based on the report of a tick bite and the presence of typical erythema migrans, which was sometimes combined with a general infection syndrome; in 42 out of 142 patients (29.6%) tested in PCR, the clinical diagnosis was confirmed by PCR detection of* B. burgdorferi* sensu lato DNA in the blood. ITBB monoinfection was diagnosed in 220 EM patients; in 89 and 18 patients, ITBB was accompanied by HGA (confirmed by PCR detection of* Anaplasma phagocytophilum* DNA in the blood and/or by recombinant ELISA detection of IgM and IgG to* A. phagocytophilum* in acute- and/or convalescent-phase serum samples) or TBE (confirmed by ELISA detection of IgM to TBE virus in sera), respectively. Most of the EM patients (93.3%) had single skin lesions which were located in the site of a tick bite, usually on the trunk and extremities. In 5 patients (2.3%) with EM monoinfection and 17 EM/HGA patients (19.1%), multiple EM skin lesions (2 to 39) appeared on days 9-16 after disease onset.

The clinical diagnosis of ITBB in EM-free patients (N = 115) was based on the report of a tick bite, presence of characteristic clinical manifestations consistent with borreliosis [5, 14], and laboratory evidence of* B. burgdorferi* sensu lato infection. The clinical diagnosis was confirmed by PCR detection of* B. burgdorferi* sensu lato DNA in the blood (in 23 out of 46 patients (50.0%) tested in PCR) and/or by recombinant ELISA detection of anti-borreliae IgM in acute- and/or convalescent-phase serum samples. ITBB monoinfection was diagnosed in 38 of EMF patients; in 44 and 33 patients, ITBB was accompanied by HGA (confirmed by PCR detection of* A. phagocytophilum* DNA in the blood and/or by recombinant ELISA detection of IgM and IgG to* A. phagocytophilum* in acute- and/or convalescent-phase serum samples) or TBE (confirmed by ELISA detection of IgM to TBE virus in sera), respectively.

All ITBB patients were treated with antibiotics (mainly doxycycline) at first visit to a clinic [5]. The patients with TBE coinfection were injected with specific antiviral immunoglobulin. Serum samples from majority of patients (88.5%) were taken two to five times (prior to treatment and at different times of observation).

### 2.4. PCR

DNA was extracted from the whole blood dried on filter paper (Whatman 903) by using the Sample-NA kit (DNA Technology, Moscow, Russia) according to manufacturer's instructions. Purified DNA isolates were frozen at -20°C. Amplification was performed in a Tercic Thermal Cycler (DNA Technology).

*B. burgdorferi* sensu lato 5S-23S rRNA intergenic spacer was amplified by using nested PCR with two primer pairs (Bb23SN1–Bb23SC1 and IGSb1–IGSa2) [24]. The PCR inhibition possibility was controlled by 150-bp long fragments of internal standard [25]. DNA isolated from* B. burgdorferi *sensu stricto B31 was used as a positive control. All positive amplicons (222–255-bp long) were purified with the Wizard PCR Preps DNA Purification System (Promega, Madison, USA) and treated by Tru1I (MseI) restriction enzyme (MBI Fermentas, Vilnius, Lithuania). PCR fragments were visualized under UV irradiation after electrophoresis in agarose gels containing ethidium bromide. The genotyping of* Borrelia* was performed by analysis of restriction fragment length polymorphism of rrf(5S)–rrl(23S) intergenic spacer amplicons [24].

*A. phagocytophilum* DNA was amplified by using nested PCR with two primer pairs (ge3a1ge10r2 and ge9f3–ge2r) targeted the 16S rRNA gene fragments [26]. DNA isolated from* A. phagocytophilum* was used as a positive control. Reaction products (546-bp long) were analyzed by agarose gel electrophoresis [27]. Only genetic variant 2 of* A. phagocytophilum* was identified in the study region previously [28].

### 2.5. ELISA

All serum samples (N = 1089) were tested for IgM and IgG to* B. burgdorferi* sensu lato,* A. phagocytophilum*, and TBE virus. Anti-borrelial IgM and IgG were detected by ELISA Omnix KS-001 IgM and KS-002 IgG (Omnix, St. Petersburg). The ELISA consisted of a mixture of recombinant antigens of three* Borrelia* genospecies (*Bb* B31,* Bg* Ip90, and* Ba* ACA-1) and thus could detect (but not discriminate) specific antibodies against any of these species. IgM and IgG to* A. phagocytophilum* were detected by recombinant ELISA Omnix KS-010 IgM and KS-011 IgG. Anti-TBE IgM was detected by the qualitative VectoTBE-IgM ELISA kit (Vector, Novosibirsk, Russia).

### 2.6. PHOSPHAN

#### 2.6.1. Control Sera, Peptide, and Recombinant Antigens

“Positive” and “negative” control serum samples as well as C6 peptides (from* Bb* B31,* Bg* Ip90, and* Ba* ACA-1) and recombinant antigens (OspC, VlsE) used in this study were described in detail in our earlier research [23]. The structure of C6 peptides and the mode of their pretreatment before adsorption in microplates were described elsewhere [29, 30].

#### 2.6.2. PHOSPHAN Performance

PHOSPHAN was performed in 96-well microplates as described in detail elsewhere [23]. In brief, an array of nine dots (three per antigen) was printed on the well bottoms. The dots contained combinations of C6* Bb *B31, OspC and VlsE (for IgM detection) or C6* Bb *B31, C6* Bg* Ip90, and C6* Ba *ACA-1 (for IgG detection). The results were expressed as values of the Lyme Index (*LI*) and were considered positive (IgM or IgG detected) at* LI* ≥ 1.

### 2.7. Statistics

Differences between proportions were considered significant at 2-tailed* P* ≤0.05 (Fisher's exact test).

## 3. Results

### 3.1. Patients

Table 1 demonstrates the clinical and epidemiological characteristics of ITBB patients (N = 442) enrolled in the research. These patients were divided into 6 groups according to the clinical form of ITBB (erythemic or erythema-free) and the presence of coinfection with HGA or TBE. The patients of these groups did not differ significantly for age, sex (except the EMF/HGA patients, among which the number of men was significantly higher than women), and time of admission after onset of the disease. The majority of patients (96.2%) mentioned a tick bite preceding the illness. Patients with the erythema-free form of ITBB became sick (after tick bite) significantly later (median: 14; 95% CI: 12; 16) than EM patients (median: 11; 95% CI: 9; 11). In the blood of EM/HGA patients and the erythema-free patients, the DNA of* B. burgdorferi* sensu lato (mainly* B. garinii*) was detected more frequently than in EM patients (mainly* B. afzelii*) (Table 1). These results are consistent with the data on a longer incubation period [3, 14] and more intensive hematogenous dissemination of* Borrelia* in the erythema-free ITBB patients [5] as well as on clinical manifestations associated with* B. garinii* or* B. afzelii* infections [13].

In the majority (more than 70%) of EM patients, including those with HGA and TBE coinfections, the course of the disease was generally mild (Table 1). Typical EM (usually with a diameter of over 10 cm) was accompanied in some cases by symptoms and signs of a general infection syndrome. Most (67 to 87%) of the erythema-free patients had a moderate course of the disease (Table 1) due to development of system and organ pathology [14].

At entry to the hospital, positive VlsE C6 antibody responses were detected in 39-73% of patients in PHOSPHAN tests for total IgM and IgG (C6 IgM/IgG); the lowest index was measured in EM/TBE patients. However, predominant IgG antibody responses to the C6 peptide were not detected in some patients (Table 1), although they were expected [31]. To understand the reasons of this discrepancy and improve the reliability of results, only 391 out of 442 (88.5%) patients were included in the following research; from each of these individuals two (or more) serum samples were collected (at the entry and at different times of recovery).

### 3.2. Sensitivity of* B. burgdorferi* C6 Peptide Based PHOSPHAN

Prior to treatment, the PHOSPHAN positivity for* Bb* C6 IgM and C6 IgG correlated directly with disease duration in all ITBB patients except the EM/TBE and EMF/TBE patients. The number of positive samples was significantly greater in patients with a longer duration of illness (≥7 days). Positive PHOSPHAN reaction for C6 IgG was observed more frequently than for C6 IgM only in EM and EM/HGA patients. In all other patients, the number of positive results with C6 in tests for IgG was about the same as for IgM. The frequency of positive reactions for total IgM/IgG exceeded that for C6 IgG alone; however these differences were not statistically significant (Table 2).

Similar patterns of reactivity were measured with C6 peptides from* B. garinii* and* B. afzelii* (data not shown). The frequency of positive reactions (for IgG and/or IgM) with these peptides and the results achieved with* Bb* C6 did not differ significantly (both at the baseline and during the period of observation) in any of the patient groups. Therefore, the results obtained with* Bg *C6 and* Ba* C6 were excluded from further consideration.

### 3.3. Detection of IgM to OspC and VlsE in PHOSPHAN

Prior to treatment, the PHOSPHAN positivity to OspC and VlsE in tests for IgM correlated directly with disease duration in EM patients as well as in EM/HGA and EMF/HGA patients. In other patients, IgM responses to recombinant proteins were negative (EM/TBE patients) or could be detected irregularly prior to treatment (EMF and EMF/TBE patients) (Table 3).

### 3.4. The Frequency of IgM and IgG to* Borrelia* Antigens Detected in Sera from ITBB Patients

Table 4 demonstrates the frequency of anti-*Borrelia* IgM and IgG antibodies detected in serum samples (prior to treatment and at convalescence) from ITBB patients. Positive PHOSPHAN reactions for C6 IgG were recorded more frequently than for C6 IgM, OspC IgM, or VlsE IgM only in EM and EM/HGA patients. Positive results with any of these antigens were detected significantly more frequently (*p* <0.05) in serum samples from EM patients with* Borrelia-Anaplasma* infection than in EM patients with* Borrelia* monoinfection. In other patients, the number of positive reactions in tests for C6 IgG and C6 IgM or C6 IgG and VlsE IgM did not differ significantly.

In general, the frequency of positive reactions for C6 IgM/IgG was higher as compared to C6 IgG. However, these differences were statistically significant only in the erythema-free patients. Additional detection of VlsE IgM increased the overall sensitivity of the C6 IgM/IgG variant; these differences were statistically significant only in EMF/HGA patients.

### 3.5. Evolution of Antibody Responses to* Borrelia* Antigens during the Disease Progression

#### 3.5.1. EM Patients

**Figure 1:**
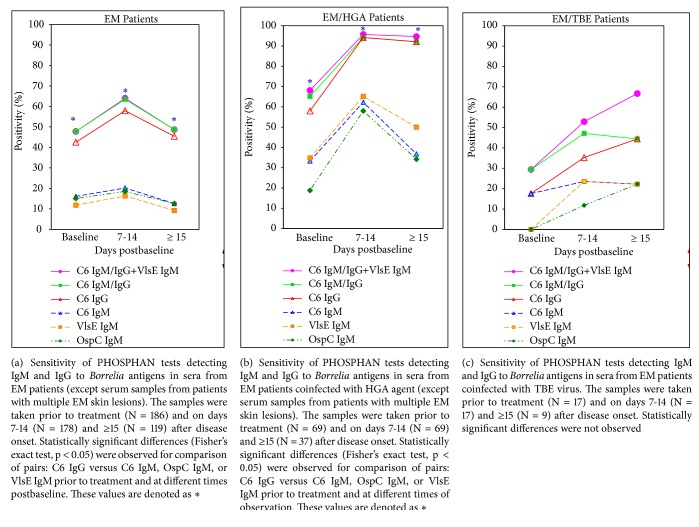


In the study of EM patients, the PHOSPHAN results confirmed our earlier observations, obtained on a significantly smaller group of EM patients [23], on the predominant contribution of C6 IgG to the total antibody responses (prior to treatment and at all-time intervals postbaseline) while the contribution of IgMs to C6, OspC, or VlsE was statistically insignificant (*p* >0.05). The frequency of detection of IgMs reacting with C6, OspC, or VlsE antigens was low ( ≤ 20%) at all times of observation (Figure 1(a)).

#### 3.5.2. EM Patients versus EM/HGA Patients

Figure 1(b) shows a similar pattern of antibody responses to* Borrelia* antigens in EM/HGA patients as compared to EM patients. At the baseline, the proportion of positive samples for C6 IgM/IgG was slightly higher than for C6-IgG; in later periods of observation, the frequency of these two variants was identical. Maximum frequency of positive responses for C6 IgG (94.2%) and for IgMs (58-65.2%) was observed on days 7-14 after start of treatment. At later time intervals, the percentage of positive samples with IgMs decreased more rapidly than with C6-IgG (Figure 1(b)).

The frequency of positive reactions for C6 IgM, C6 IgG, and VlsE IgM in the EM/HGA patients was significantly (*p* <0.05) higher than in EM patients (both at the baseline and at all times of recovery); differences in the frequency of OspC IgM detection in EM/HGA and EM patients were not statistically significant.

#### 3.5.3. EM Patients versus EM/TBE Patients

Figure 1(c) illustrates a delay in development of the antibody responses in EM/TBE patients. Prior to treatment, the frequency of positive reactions for C6 IgM and C6 IgG was equally low (17.6%) while the results with OspC and VlsE in tests for IgM were negative. During the disease progression, the frequency of positive results with the C6 peptide in tests for IgG was gradually increased (up to 44%). IgMs to OspC and VlsE were detected on days 7-14 and later periods; the frequency of positive reactions to these antigens did not exceed 22%.

In general, the number of positive C6 IgM/IgG responses was insignificantly higher than of C6 IgG alone. Additional detection of VlsE IgM at later time intervals (≥ 15 days) slightly increased the overall sensitivity of the C6 IgM/IgG tests up to 67% (Figure 1(c)).

Differences in the frequency of positive reactions to any of* Borrelia* antigens in EM/TBE and EM patients lacked statistical significance at all-time intervals of observation.

#### 3.5.4. EM Patients versus Erythema-Free Patients

**Figure 2:**
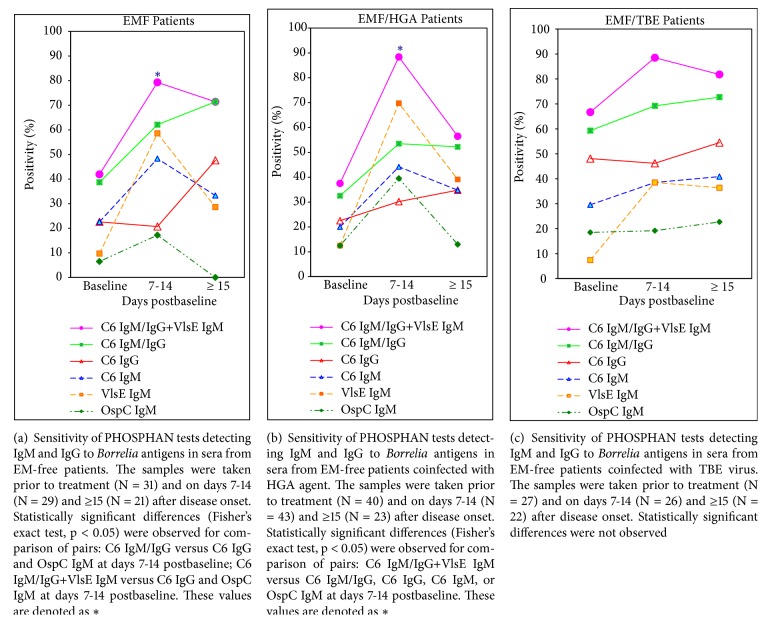


Figure 2(a) shows a different pattern of development of the antibody responses in the erythema-free patients as compared to EM patients. At the baseline, the number of positive results with C6 in tests for IgM and IgG was equally low. On days 7-14 after start of treatment, the number of positive samples with C6 IgG remained the same while the number of positive sera with C6 IgM increased up to 48.3% (*p* >0.05); later on (≥ 15 days), the number of positive reactions for C6 IgG increased up to 48%. The proportion of positive C6 IgM/IgG responses was greater than for IgG alone but the difference was statistically significant only in samples collected on days 7-14 after start of treatment. These data provide evidence for delay in development of IgG antibody responses to the peptide C6 in the EMF patients; this delay is accompanied by simultaneous accumulation of positive C6 IgM and VlsE IgM responses on days 7-14 postbaseline.

In general, positive IgM responses were detected in 6.5–23% samples from the EMF patients prior to treatment. Maximum frequency of IgMs detection (17.2-58.6%) was observed on days 7-14 after start of treatment; the maximum value of this parameter was measured for VlsE IgM (the difference was statistically significant for comparison of VlsE IgM versus C6 IgG). The frequency of positive reactions for C6 IgM/IgG (62.1%) in this time interval was significantly higher than for C6 IgG (20.7%) due to significant contribution of the C6 IgM. Additional detection of VlsE IgM on days 7-14 after start of treatment increased the overall sensitivity of the C6 IgM/IgG test up to 79.3% (Figure 1(c)); however, the difference lacked statistical significance.

The frequency of positive reactions to C6 in tests for IgG was significantly (*p* <0.05) higher (at the baseline and on days 7-14 after treatment) in EM patients as compared to the erythema-free ITBB patients. In contrast, the proportion of positive C6 IgM and VlsE IgM responses was significantly (*p* <0.05) higher in the erythema-free patients (at all-time intervals after start of treatment) than in EM patients.

#### 3.5.5. EMF Patients versus EMF/HGA Patients

Figure 2(b) shows a similar pattern of development of the antibody responses in EMF/HGA patients as compared to EMF patients. In general, the proportion of positive C6 IgG and C6 IgM responses was comparable both at the baseline (20.0-22.5%) and at all-time intervals postbaseline. The number of positive C6 IgM/IgG responses exceeded that for C6 IgG at all times of observation; however, the difference lacked statistical significance.

The maximum number of positive responses for IgMs (39.5-69.8%) was observed on days 7-14 after start of treatment; the maximum value of this parameter was measured for VlsE IgM (the difference was statistically significant for comparison of VlsE IgM versus C6 IgG). Additional detection of VlsE IgM significantly increased the overall sensitivity of the C6 IgM/IgG test (*p* <0.05).

#### 3.5.6. EMF Patients versus EMF/TBE Patients

Figure 2(c) shows a similar pattern of development of the antibody responses in EMF/TBE patients as compared to EMF (Figure 2(a)); EMF/HGA (Figure 2(b)) and EM/TBE patients (Figure 1(c)).

The frequency of positive results with the peptide C6 in tests for IgG was slightly increasing during the disease progression. The proportion of positive C6 IgM/IgG responses exceeded that for C6-IgG at all times of observation; however, the difference lacked statistical significance. Additional detection of VlsE IgM on days 7-14 postbaseline increased the overall sensitivity of the C6 IgM/IgG test up to 89% (*p* >0.05).

Differences in the frequency of positive reactions to any of* Borrelia* antigens in EMF/TBE and EMF patients lacked statistical significance at all times of observation.

## 4. Discussion

In the present study, we applied a recently developed method of multiplex phosphorescence analysis to detect specific antibody responses to a number of* Borrelia *antigens. The advantages of PHOSPHAN method as compared to ELISA and immunoblot techniques were described in detail elsewhere [23].

Serum samples for this study were collected in Perm Krai, which is one of the highly endemic regions of Russia. According to laboratory testing of approximately 500 patients from this area, who became ill during the period of seasonal activity of ticks in 2010, the disease with ITBB was confirmed in 45% of cases; TBE (10.4%), HGA (5%), and human monocytic ehrlichiosis (1.7%) cases were less frequent; mixed infections were identified in 27% of patients [32]. These data provide evidence for high pretest probability of ITBB in this region, in combination with other tick-borne infections in particular. In the study region, no research has been performed on the prevalence of* B. miyamotoi* in tick and human samples, which could confirm a possible role of this spirochete in etiology of ITBB in EMF patients.

One of the most promising approaches to early serologic diagnosis of ITBB caused by* B. burgdorferi* sensu lato is based on the use of the peptide C6 from VlsE protein of* B. burgdorferi* [31]. The commercial test system (Immunetics C6 ELISA, USA) detects total IgM and IgG antibodies to this peptide, although the main contribution is provided by IgG [31]. We confirmed the effectiveness of this approach in EM patients in Russia [23]. In the present study, we compared the efficiency of the C6 peptide based PHOSPHAN for serologic confirmation of ITBB in Russian patients with EM and the erythema-free manifestations of the disease, which was accompanied in some patients by the ongoing coinfections with HGA or TBE.

Prior to treatment, the frequency of positive results to* Bb* C6 in tests for total IgM/IgG antibodies did not differ significantly in the patient groups. The PHOSPHAN positivity correlated directly with disease duration in all patients except the EM/TBE and EMF/TBE patients (Table 2). Total frequency of positive reactions to the peptide C6 at different times of recovery did not differ significantly in the groups of patients; however, the values of this parameter were greater in EM/HGA patients, and the lowest values were observed in EM/TBE patients (Table 4, Figures 1 and 2). These results are comparable to the C6 ELISA data [33] and confirm the efficiency of* Bb* C6 IgM/IgG PHOSPHAN assay for serologic confirmation of ITBB in EM and EMF patients early after disease onset.

However, the contribution of IgM and IgG to the total antibody response to C6 differed significantly in the patient groups.

“Typical” IgM and IgG antibody responses to the peptide C6 [31] were observed only in EM and EM/HGA patients, prior to treatment (Table 2) and at different times of recovery (Table 4, Figures 1(a) and 1(b)). Positive C6 IgG responses were detected significantly more frequently than C6 IgM. Detection of OspC IgM or VlsE IgM did not improve significantly the overall sensitivity of C6 IgM/IgG test, which is consistent with previous data [23, 34].

The frequency of positive antibody responses to* Borrelia* antigens was significantly greater in EM/HGA patients than in EM patients (Table 4, Figures 1(a) and 1(b)). These data, together with positive* B. burgdorferi* sensu lato DNA PCR results in almost 70% of EM/HGA patients (Table 1), allowed us to suggest that the presence of intracellular infection did not limit the spirochete dissemination.

The intensity of antibody responses to* Borrelia *antigens was also significantly greater in EM/HGA patients. Median* LI* values for C6 IgG were 32.3 (95% CI: 12.9; 65.9) versus 7.4 (95% CI: 4.5; 10.9) in EM/HGA and EM patients with negative* B. burgdorferi* sensu lato DNA PCR (p < 0.05), and 66.6 (95% CI: 53; 96) versus 8.8 (95% CI: 3.3; 52.5) in EM/HGA and EM patients with positive PCR result (p < 0.05). These data provide evidence for a stronger antigenic stimulation of the immunocompetent cells, responsible for production of specific antibodies to* Borrelia* antigens in EM/HGA patients, and indirectly confirm the spirochete accumulation in the blood of patients with* Borrelia-Anaplasma* infection, which is consistent with results obtained in the animal models [35–37]. As compared to EM patients with* Borrelia* monoinfection, clinical manifestations of* Borrelia-Anaplasma* infection were characterized by a more severe general infection syndrome which was accompanied in some cases by pathology of the internal organs (the liver or less frequently the kidney) [38].

In EM/TBE patients, the proportion of positive C6 IgM and C6 IgG results (prior to treatment) was equally low (Table 2), while the OspC IgM and VlsE IgM antibody responses to OspC and VlsE were negative (Table 3). During disease progression, the frequency of positive reactions to* Borrelia* antigens gradually increased (Figure 1(c)). Although the number of serum samples at different times of recovery was rather small for making a reliable conclusion, the data obtained confirmed the delay in development of the antibody responses to* Borrelia *antigens in patients with* Borrelia*-TBE infection, which also has been noted by other authors; it is believed that the dual infection has no effect on the antibody production against the virus (for review [7]). Active accumulation of IgM to TBE virus in the debut of illness with tick-borne encephalitis [39] as well as the introduction of antiviral immunoglobulin to TBE patients can possibly affect the development of antibody responses to C6 and other* Borrelia* antigens. Clinical manifestations of* Borrelia*-TBE virus infections are usually characterized by more acute onset as compared to ITBB patients and more frequent symptoms of general infection syndrome as compared to TBE patients (for review [7]).

In the erythema-free ITBB patients, those with dual* Borrelia-Anaplasma* and* Borrelia*-TBE virus infections included, we observed atypical antibody responses to* B. burgdorferi* sensu lato antigens as compared to EM patients. The delay in development of IgG antibody responses to C6 was followed by active accumulation of IgMs to C6, OspC, and VlsE in convalescent-phase serum samples (Figures 2(a), 2(b), and 2(c)). The frequency of positive C6 IgM and C6 IgG reactions (prior to treatment) was comparable (Table 2); however, on days 7-14 postbaseline, the contribution of C6 IgM to the total C6 IgM/IgG antibody response was greater than of C6 IgG (Figures 2(a), 2(b), and 2(c)). Thus, the sensitivity of C6 IgM/IgG PHOSPHAN assay was significantly higher than of C6 IgG alone (Table 4). The maximum number of positive responses for IgMs, to protein VlsE in particular, was observed on days 7-14 after start of treatment (Figures 2(a), 2(b), and 2(c)); additional detection of VlsE IgM increased the overall sensitivity of the C6 IgM/IgG test.

These results provide evidence for modulation of the antibody responses in the erythema-free patients. A significant increase in the level of immunoglobulin M was observed at no change in concentrations of immunoglobulin G and in the cellular component of the immunity [5]. Significant predominance of the IgM response to* B. burgdorferi* and the failure of B cells to undergo class-switch recombination to an IgG response was demonstrated previously in experimental animal models [40]. Importantly, in EMF patients with PCR confirmed* B. burgdorferi* sensu lato infection, the frequency of positive antibody responses to* Borrelia* antigens was comparable with the values shown in Table 4, except the fact of more frequent detection of C6 IgG (50-59% versus 28.3-49.3%). Additional detection of VlsE IgM increased the overall sensitivity of the C6 IgM/IgG test up to 76-88%. These data possibly indicate an important role of IgM antibodies in elimination of the pathogen from the blood stream of EMF patients, which was demonstrated in experimental animal models [40].

We can also speculate that at least a part of EMF patients suffered from the ITBB disease caused by* B. miyamotoi *instead of* B. burgdorferi* sensu lato; in patients with PCR confirmed presence of* B. burgdorferi* sensu lato DNA, mixed infection with both agents could occur. This hypothesis, however, is supported only by atypical antibody responses to* B. burgdorferi* sensu lato antigens, which were previously discovered in patients with* B. miyamotoi* disease [15, 17, 18, 22]. Importantly, the* B. miyamotoi*'s status as a pathogen has only recently been established. There is still no an adequate and appropriate immunocompetent animal model to study* B. miyamotoi* infection and identify characteristic symptoms and pathologies of the BMD. The symptoms of this disease are interpreted and extrapolated from complex human cases where disease pathology can be complicated by underlying or unreported medical conditions or coinfections [41].

We can also hypothesize that the erythema-free forms of borreliosis may be caused by other tick-transmitted species of* Borrelia*, both new and already described, whose role in the etiology of the disease has not yet been adequately proven. Serology developed for Lyme borreliosis can help to confirm an active infection in such patients which provided the cross-reactivity between the detected antibodies and* B. burgdorferi* sensu lato antigens [42] which allows for in-time treatment with antibiotics.

The DNA of* B. garinii* was detected in the blood of ITBB patients (except EM patients with ITBB monoinfection) more frequently than* B. afzelii* (Table 1). However, positive antibody responses were recorded more frequently with* Bb* C6 or* Ba* C6 than with* Bg* C6 as was shown previously for EM patients [23]. These data confirm that the IR6 region of* Bb *VlsE is highly conserved among European pathogenic genospecies of* B. burgdorferi* sensu lato [43].

Data on the timing of production of OspC IgM and VlsE IgM, compared to C6 IgM and C6 IgG, in the EM-free patients, as well as in EM and EMF patients with the ongoing HGA and TBE coinfections, are presented here for the first time. Strong C6 IgG and VlsE IgM antibody responses can be considered as important markers of an active* Borrelia* infection in EM and the erythema-free ITBB patients, respectively.

The limitation of our study was the fact that the hypothesis on probable* B. miyamotoi* disease in EMF patients was based just on antibody profile similarity with previously published data. No serologic or PCR tests were performed to confirm this hypothesis.

## 5. Conclusion

The multiplex PHOSPHAN is a promising method for detecting IgM and IgG antibody responses to a number of* Borrelia *antigens. The detection of* Bb* C6 IgM/IgG provides effective serologic confirmation of ITBB in both EM patients and the erythema-free patients early after disease onset. As the* Borrelia* infection progressed in the erythema-free patients, the* B. burgdorferi* C6 IgM/IgG test needs to be supplemented with detection of VlsE IgM. In general, the PHOSPHAN positivity was recorded in 240 of 293 (81.9%) patients with erythema migrans and 85 of 98 (86.7%) patients without cutaneous manifestations of the disease. PHOSPHAN provided serologic confirmation of the disease in 59 of 65 (90.8%) patients, whose blood contained* B. burgdorferi* sensu lato DNA; only 6 patients tested positive in PCR (5 with EM and 1 without this skin manifestation) were seronegative. The observations from this study document significant differences in the repertoire and kinetics of antibody responses to* Borrelia* antigens in ITBB patients depending on clinical manifestations (erythemic or erythema-free) of the disease and the presence of coinfection with HGA or TBE agents. Further work is needed to determine the interplay between the antibody responses to* Borrelia* antigens, primarily to C6 peptide and the parent VlsE molecule, and the underlying mechanisms of innate and adaptive immune responses in both EM and EM-free patients. We also plan to test a possible role of* B. miyamotoi* in etiology of ITBB disease in EMF patients by using the PHOSPHAN immunoassay supplemented with GlpQ protein specific to this pathogen.

## Figures and Tables

**Table 1:** Clinical and epidemiological characteristics of ITBB patients (N = 442) enrolled in the research.

Characteristic	EM Patients			EMF Patients		
	Monoinfection (N = 220)	Coinfection with HGA (N = 89)	Coinfection with TBE (N = 18)	Monoinfection (N = 38)	Coinfection with HGA (N = 44)	Coinfection with TBE (N = 33)
Median (range) age (yr)	58 (15-87)	55 (18-84)	60 (25-80)	53 (16-86)	54 (15-81)	53 (18-79)
Sex						
No. (%) male	102 (46.4)	48 (53.9)	8 (44.4)	18 (47.4)	31 (70.5)	15 (45.5)
No. (%) female	118 (53.6)	41 (46.1)	10 (55.6)	20 (52.6)	13 (29.5)	18 (54.5)
Tick bite history						
No. (%) of patients	216 (98.2)	84 (94.4)	18 (100)	34 (89.5)	43 (97.7)	30 (90.9)
Median (range) time from tick bite to disease (days)	11 (1-65)	11 (2-34)	10 (1-29)	14 (1-50)	14 (2-59)	15 (1-50)
Median (range) time from disease to study entry (days)	5 (1-61)	7 (1-48)	3 (1-25)	4 (1-63)	4 (1-62)	4 (1-49)
Disease severity						
No. (%) mild	178 (80.9)	64 (71.9)	15 (83.3)	5 (13.2)	7 (15.9)	11 (33.3)
No. (%) moderate	42 (19.1)	25 (28.1)	3 (16.7)	33 (86.8)	37 (84.1)	22 (66.7)
No. (%) of patients with positive *Borrelia* PCR _ _^(a)^	13/93 (13.9)	26/38 (68.4)	3/11 (27.3)	6/9 (66.7)	12/20 (60.0)	5/17 (29.4)
including no. of patients with						
* B. afzelii*	7	1	1	0	2	0
*B. garinii*	4	16	2	6	7	5
*B. afzelii* + *B. garinii*	0	5	0	0	2	0
*B. burgdorferi* sensu lato	2	4	0	0	1	0
No. (%) of patients with positive VlsE C6 antibody response at study entry _ _^(b)^						
C6 IgM/IgG	94 (42.7)	65 (73.0)	7 (38.9)	18 (47.4)	19 (43.2)	24 (72.7)
C6-IgM	36 (16.4)	34 (38.2)	4 (22.2)	11 (28.9)	11 (25.0)	8 (24.2)
C6-IgG	80 (36.4)	56 (62.9)	5 (27.8)	8 (21.1)	12 (27.3)	19 (57.6)

EM = erythema migrans; EMF = erythema migrans-free. Data were determined by the hospital's medical laboratory. Disease severity was determined as a function of a number and intensity of the associated symptoms. ^(a)^ Number of patients tested positive (numerator) out of total number of patients tested by *Borrelia* PCR (denominator). ^(b)^ Results of *B. burgdorferi* C6 peptide based PHOSPHAN are represented. Statistically significant differences (Fisher's exact test, *p* < 0.05) were observed for comparison of median time from tick bite to disease in EM patients (monoinfection) versus any of the EM-free patients.

**Table 2:** Sensitivity of *B. burgdorferi* C6 peptide based PHOSPHAN tests for serum IgM and IgG antibody detection in samples from ITBB patients prior to treatment (N = 391).

Patient Group	Number (%) of positive serum samples at the baseline in ITBB patients as a function of disease duration							
	<7 days				≥ 7 days			
	No. of patients	C6 IgM	C6 IgG	C6 IgM/IgG	No. of patients	C6 IgM	C6 IgG	C6 IgM/IgG
EM patients	133	16 (12.0)	50 (37.6)	56 (42.1)	58	16 (27.6)	30 (51.7)	35 (60.3)
EM/HGA patients	42	13 (31.0)	21 (50.0)	23 (54.8)	43	19 (44.2)	31 (72.1)	34 (79.1)
EM/TBE patients	11	2 (18.2)	2 (18.2)	3 (27.3)	6	1 (16.7)	1 (16.7)	2 (33.3)
EMF patients	24	5 (20.8)	5 (20.8)	8 (33.3)	7	2 (28.6)	2 (28.6)	4 (57.1)
EMF/HGA patients	31	3 (9.7)	4 (12.9)	6 (19.4)	9	6 (66.7)	5 (55.6)	7 (77.8)
EMF/TBE patients	19	7 (36.8)	8 (42.1)	11 (57.9)	8	1 (12.5)	5 (62.5)	5 (62.5)

EM = erythema migrans, EMF = erythema migrans-free, EM/HGA = EM patients coinfected with HGA agent, EM/TBE = EM patients coinfected with TBE virus, EMF/HGA = EMF patients coinfected with HGA agent, and EMF/TBE = EMF patients coinfected with TBE virus. Statistically significant differences (Fisher's exact test, *p* < 0.05) were observed for comparison of pairs: C6 IgM versus C6 IgG in EM patients at EM duration <7 days and ≥7 days; C6 IgM versus C6 IgG in EM/HGA patients at EM duration ≥7 days; C6 IgM at disease duration <7 days versus C6 IgM at disease duration ≥7 days in EMF/HGA patients; C6 IgG at disease duration <7 days versus C6 IgG at disease duration ≥7 days in EMF/HGA patients. All other comparisons were not statistically significant.

**Table 3:** IgM antibody responses to OspC and VlsE in PHOSPHAN prior to treatment (N = 391).

Patient Group	Number (%) of positive serum samples at the baseline in ITBB patients as a function of disease duration					
	<7 days			≥ 7 days		
	No. of patients	OspC IgM	VlsE IgM	No. of patients	OspC IgM	VlsE IgM
EM patients	133	13 (9.8)	13 (9.8)	58	15 (25.9)	9 (15.5)
EM/HGA patients	42	8 (19.0)	10 (23.8)	43	17 (39.5)	24 (55.8)
EM/TBE patients	11	0	0	6	0	0

EMF patients	24	2 (8.3)	2 (8.3)	7	0	1 (14.3)
EMF/HGA patients	31	0	2 (6.5)	9	4 (44.4)	3 (33.3)
EMF/TBE patients	19	5 (26.3)	1 (5.3)	8	0	1 (12.5)

EM = erythema migrans, EMF = erythema migrans-free, EM/HGA = EM patients coinfected with HGA agent, EM/TBE = EM patients coinfected with TBE virus, EMF/HGA = EMF patients coinfected with HGA agent, and EMF/TBE = EMF patients coinfected with TBE virus. Statistically significant differences (Fisher's exact test, *p* < 0.05) were observed for comparison of pairs: OspC IgM at EM duration <7 days versus OspC IgM at EM duration ≥7 days in EM patients; OspC IgM at disease duration <7 days versus OspC IgM at disease duration ≥7 days in EMF/HGA patients; VlsE IgM at disease duration <7 days versus VlsE IgM at disease duration ≥7 days in EMF/HGA patients; OspC IgM at EM duration ≥7 days in EM patients versus OspC IgM at disease duration ≥7 days in EMF/HGA patients; VlsE IgM at EM duration ≥7 days in EM patients versus VlsE IgM at disease duration ≥7 days in EMF/HGA patients. All other comparisons were not statistically significant.

**Table 4:** Frequency of positive antibody responses to* Borrelia* antigens in serum samples (N = 1018) from ITBB patients.

Patient Group	No. of sera	Number (%) of positive sera (at the baseline and convalescent) to* Borrelia* antigens					
		OspC IgM	VlsE IgM	C6 IgM	C6 IgG	C6 IgM/IgG	C6 IgM/IgG + VlsE IgM
EM patients	498	78 (15.7)	65 (13.1)	87 (17.5)	241 (48.4)	266 (53.4)	267 (53.6)
EM/HGA patients	215	95 (44.2)	117 (54.4)	101 (47.0)	172 (80.0)	177 (82.3)	182 (84.7)
EM/TBE patients	43	4 (9.3)	6 (14.0)	9 (20.9)	13 (30.2)	17 (39.5)	20 (46.5)

EMF patients	81	7 (8.6)	26 (32.1)	28 (34.6)	23 (28.4)	45 (55.6)	51 (63.0)
EMF/HGA patients	106	24 (22.6)	44 (41.5)	36 (34.0)	30 (28.3)	48 (45.3)	66 (62.3)
EMF/TBE patients	75	15 (20.0)	20 (26.7)	27 (36.0)	37 (49.3)	50 (66.7)	59 (78.7)

EM = erythema migrans, EMF = erythema migrans-free, EM/HGA = EM patients coinfected with HGA agent, EM/TBE = EM patients coinfected with TBE virus, EMF/HGA = EMF patients coinfected with HGA agent, and EMF/TBE = EMF patients coinfected with TBE virus. Statistically significant differences (Fisher's exact test, p < 0.05) were observed for comparison of pairs: C6 IgG versus OspC IgM, VlsE IgM, and C6 IgM in EM patients; C6 IgG versus OspC IgM, VlsE IgM, and C6 IgM in EM/HGA patients; C6 IgG versus OspC IgM in EMF patients; C6 IgG versus OspC IgM and VlsE IgM in EMF/TBE patients; C6 IgM/IgG versus C6 IgG in EMF/HGA patients; C6 IgM/IgG versus C6 IgG in EMF/TBE patients; C6 IgM/IgG+VlsE IgM versus C6 IgM/IgG in EMF/HGA patients; C6 IgG in EM/HGA patients versus C6 IgG in other groups of patients; C6 IgM in EM/HGA patients versus C6 IgM in EM patients and EM/TBE patients; C6 IgM/IgG in EM/HGA patients versus C6 IgM/IgG in other groups of patients. All other comparisons were not statistically significant.
